# Fabrication and Bonding of Refractive Index Matched Microfluidics for Precise Measurements of Cell Mass

**DOI:** 10.3390/polym13040496

**Published:** 2021-02-05

**Authors:** Edward R. Polanco, Justin Griffin, Thomas A. Zangle

**Affiliations:** 1 Department of Chemical Engineering, University of Utah, Salt Lake City, UT 84112, USA; eddiepolanco@chemeng.utah.edu (E.R.P.); justin.griffin@utah.edu (J.G.); 2 Huntsman Cancer Institute, University of Utah, Salt Lake City, UT 84112, USA

**Keywords:** quantitative phase imaging, biomedical microfluidics, microscopy, NOA1348, refractive index matching

## Abstract

The optical properties of polymer materials used for microfluidic device fabrication can impact device performance when used for optical measurements. In particular, conventional polymer materials used for microfluidic devices have a large difference in refractive index relative to aqueous media generally used for biomedical applications. This can create artifacts when used for microscopy-based assays. Fluorination can reduce polymer refractive index, but at the cost of reduced adhesion, creating issues with device bonding. Here, we present a novel fabrication technique for bonding microfluidic devices made of NOA1348, which is a fluorinated, UV-curable polymer with a refractive index similar to that of water, to a glass substrate. This technique is compatible with soft lithography techniques, making this approach readily integrated into existing microfabrication workflows. We also demonstrate that this material is compatible with quantitative phase imaging, which we used to validate the refractive index of the material post-fabrication. Finally, we demonstrate the use of this material with a novel image processing approach to precisely quantify the mass of cells in the microchannel without the use of cell segmentation or tracking. The novel image processing approach combined with this low refractive index material eliminates an important source of error, allowing for high-precision measurements of cell mass with a coefficient of variance of 1%.

## 1. Introduction

Microfluidics is a maturing field with broad applications ranging from chemical detection [1] to drug discovery [2]. Microfluidics has also made important contributions to biomedical research [3] such that biomedical microfluidics is an important tool for applications such as monitoring the extracellular environment [4], generating resource gradients [5], and many strategies for single cell analysis [6,7]. Many microfluidic techniques employ the optical properties of the fabrication material such as optical trapping [8], optical chemical sensing [9], or studying drug delivery in the tumor microenvironment [10].

Traditional materials used in the fabrication of microfluidic devices, such as glass or polydimethysiloxane (PDMS). The use of PDMS is especially common because the fabrication and assembly protocols are well developed, and for the two most common PDMS products, Sylgard 184 by Dow Corning and RTV 615 by Bayer Silicones, the physical properties are well characterized [11]. PDMS is also compatible with most soft lithography techniques and uses reagents and processes that are readily available to most labs. One important drawback to these materials is their high refractive index relative to water, resulting in artifacts at the interface between microfabricated structures and aqueous solutions. This causes ambiguity in measurements made near material interfaces using quantitative microscopy techniques such as localizing fluorescent signals or quantitative phase imaging (QPI) [12].

QPI is a technique that measures the phase shift of light as it passes through a transparent sample such as a microchannel structure or live cells. The measured phase shift is linearly related to the mass of a cell, making it highly relevant for studying cell growth in response to biological perturbations in the context of drug discovery or disease diagnosis [13,14]. By measuring the phase shift of light as it passes through structures in the field of view, QPI measures the integrated difference in refractive index between the different materials through the optical path. This quantity is called the optical path length. If the difference in optical path length is large (such as in the case of typical depth PDMS channels and aqueous media), then this results in a phase shift that is larger than a wavelength of light, causing ambiguities in the measurement due to the periodicity of light. This limits the applicability of microfluidics to QPI due to the importance of water as a solvent for many microfluidic applications, including biomedical microfluidics.

One solution is to increase the refractive index of the aqueous medium by tuning with an additive such as a high mass polysaccharide, increasing the refractive index of the solution [7]. However, this comes at the cost of changing the composition of cell culture medium optimized for the growth of cells in culture and is less ideal for live cell imaging during long experiments. Alternatively, fluorinated polymers with a similar refractive index matched to that of water have been shown to eliminate artifacts arising from optical measurements in aqueous media in the presence of microfabricated structures, such as microchannels and microwells [12,15,16,17,18]. However, the most important challenge for using low refractive index polymers for microfabrication is that fluorination creates materials that are notoriously nonadherent and chemically stable. This makes it challenging to seal index-matched microfluidic devices to a substrate in order to establish steady fluid flow through the device. Typical device operation reported previously requires clamps, increasing the likelihood of breaking the substrate or using an ultraviolet (UV)-curable adhesive layer, which if applied incorrectly can clog the channel during the fabrication process [15].

Previous studies of UV-curable adhesives made by a specialty manufacturer, Norland Optical Adhesives (NOA), have demonstrated the usefulness of UV-curable polymers for microfluidics and nanofluidics. For example, molds made using NOA63 were shown to be readily fabricated using a UV oven, and they could readily reproduce high aspect ratio nanofluidic channels with high precision [16,19]. This material can also be readily hydrophilized for use with aqueous solutions in microfluidics [19,20,21]. Such devices allow for flow using only capillary action without the need for an external energy source to drive flow [20,21].

Another UV curable polymer that has been shown to have important applications is NOA81, which has useful applications for creating films with microfluidic channels called microfluidic stickers. This process was used to bond microchannels to a glass substrate [22]. This technique takes advantage of the porosity of PDMS to allow oxygen to inhibit the polymerization on the surface of the material, creating a thin layer of uncured polymer. After pressing the microchannel against a glass substrate, the uncured polymer is cured in an anaerobic environment, causing the film to adhere to the glass substrate [22]. It has been shown that the surface properties of this material can also be readily modified so that it can have a wide degree of applications to both hydrophilic and hydrophobic solutions [23], as well as being compatible with electrowetting for carefully controlling aqueous wettability while maintaining a hydrophobic surface for digital microfluidics [24]. NOA81 also readily adheres to wet substrates such as glass slides or cover slips, such that live adherent cells can be studied in a millifluidic environment without having to seed and grow the cells in the channel, preserving the tissue organization for studying multicellular systems [25]. This approach omits the use of mechanical force, allowing the technique to be applied to thin substrates such as coverslips, making this approach compatible with high numerical aperture (NA) objective lenses, which are often limited in application due to the short working distance characteristic of high NA objectives.

Here, we demonstrate the assembly of an NOA1348 microfluidic sticker that can be laminated to a glass substrate. NOA1348 is a fluorinated polymer with a low polarizability, resulting in a low refractive index similar to that of water (*n* = 1.33). Previous work with this material has demonstrated that it can be used as a low refractive index medium for constructing endoscopic probes for deep tissue imaging for disease diagnosis in the brain, esophagus, or the coronary artery [26]. Here, we show that NOA1348 is compatible with soft lithography techniques and is curable in a UV oven, making it readily integrated into existing microfluidic fabrication workflows. Therefore, this method provides a reproducible and biocompatible method to fabricate microfluidic devices with the refractive index matched between the substrate and aqueous cell culture media. We describe the fabrication method and demonstrate its application to single cell trapping structures. Finally, we show that NOA1348 devices constructed using our method have 1.8% temporal and 4.2% spatial repeatability. This enables applications of this device and fabrication method to applications requiring high-precision optical measurements.

## 2. Materials and Methods

### 2.1. Master Mold Fabrication

**Figure 1:**
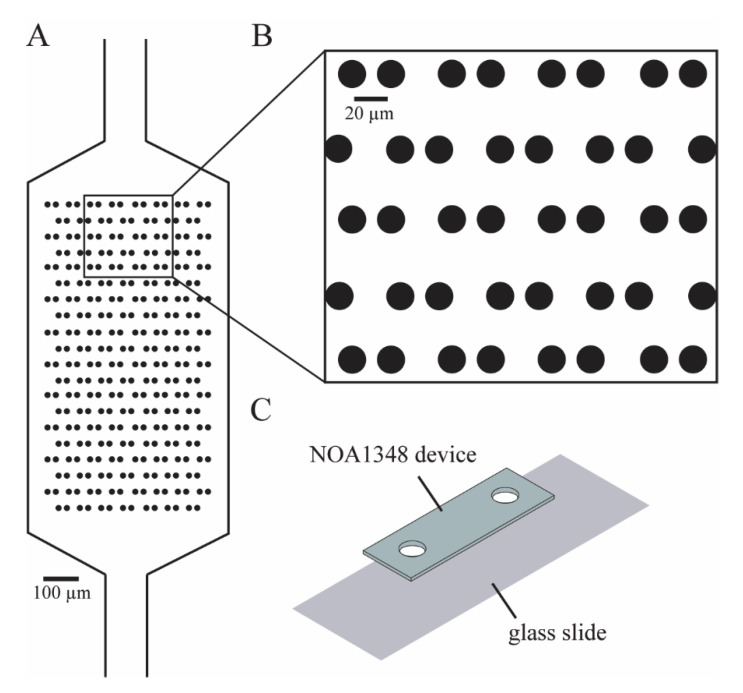
Device overview. (**A**) Microchannel uses flow to trap cells and prevent cells from attaching directly next to each other. Traps are made from NOA1348, which is a material with a refractive index similar to water (*n* = 1.33). (**B**) Trap geometry allows the media to flow through the traps to direct cells to empty traps. (**C**) Diagram summarizes the fabrication of this device, showing that there are only two layers that are pressed together to form the device.

We designed our microfluidic devices using Autocad (Autodesk, San Rafael, CA, USA) as shown in Figure 1A, such that we could maximize the number of cells caught in the cell trap array. The enlarged traps in Figure 1B shows that each trap consists of two identical cylinders (circular cross-section shown), with a small gap in between them to allow flow to direct cells toward the empty traps. We imported the Autocad design (see Supplementary Materials) into a high-resolution photolithography mask pattern generator (Heidelberg 101, Heidelberg Instruments, Heidelberg, Germany) in the University of Utah Nanofab in order to directly write the pattern for the microchannels onto the photoresist, which was then developed to make a mask. To make the SU-8 master molds on a 4-inch silicon wafer, we first diluted SU-8 50-100 negative photoresist (MicroChem Laboratories, Round Rock, TX, USA) with a 20:1 mass ratio of photoresist to cyclopentanone to thin out the SU-8. We spinned the photoresist onto the wafer at 2500 RPM for 1 min to create a film of 10 μm nominal (9.8 μm measured) thickness. Then, we used contact lithography to expose the photoresist with 280 mJ/cm^2^ of UV light, prior to developing the SU-8. Then, the thickness of the film was confirmed using a confocal scanning microscope (Olympus Corporation, Shinjuku, Japan). Finally, the SU-8 master was treated with trichloro(1*H*,1*H*,2*H*,2*H*-perfluorooctyl)silane (PFOTS) (Sigma-Aldrich, St. Louis, MO, USA) to facilitate the release of molded PDMS from the fine trap structures.

### 2.2. Validation of Microchannel SU-8 Mold Dimensions 

To validate the height of the microchannel, we used a commercially available Olympus LEXT confocal laser scanning microscope (Olympus Corporation, Shinjuku, Japan) to measure the step height from the silicon wafer to the top of the fabricated SU-8 master mold. This microscope measures step height by finely moving the objective lens in the z-direction (as small as 10 nm), in order to determine the height at which it achieves the highest intensity at each pixel. By recording the height at which the maximum intensity was measured at each pixel, the microscope measures the step height of the microchannel. This tool also measures the lateral dimensions of the microchannel features by measuring the pixel distance between the step measurements on each side of the microchannel.

### 2.3. Validation of PDMS and NOA Dimensions Using Brightfield Microscopy

We validated final PDMS and NOA1348 device dimensions using brightfield imaging after calibrating the distance per pixel with a stage micrometer (1 mm with 10 μm divisions).

### 2.4. PDMS Mold Fabrication

We used the SU-8 master to make a PDMS mold from which many NOA1348 devices could be made. To make our PDMS molds, we first molded the PDMS (Sylgard 184) on the SU-8 master to make a negative of the master. This negative is a replicate of the microchannel and can be used to make a mold for the microchannel. We trim the negative and treat it with PFOTS overnight before casting the mold. To make the mold, we first make a PDMS square that is 50 mm × 50 mm × 1.5 mm and cut a 10 mm × 20 mm rectangle out of the center of it. We place this over the negative so that the microchannel is centered in the cut-out rectangle and fill it with PDMS. Then, we pressed a slide on it to flatten it and cure it in a 65 °C oven for 3 h to cure.

### 2.5. Device Fabrication

**Figure 2:**
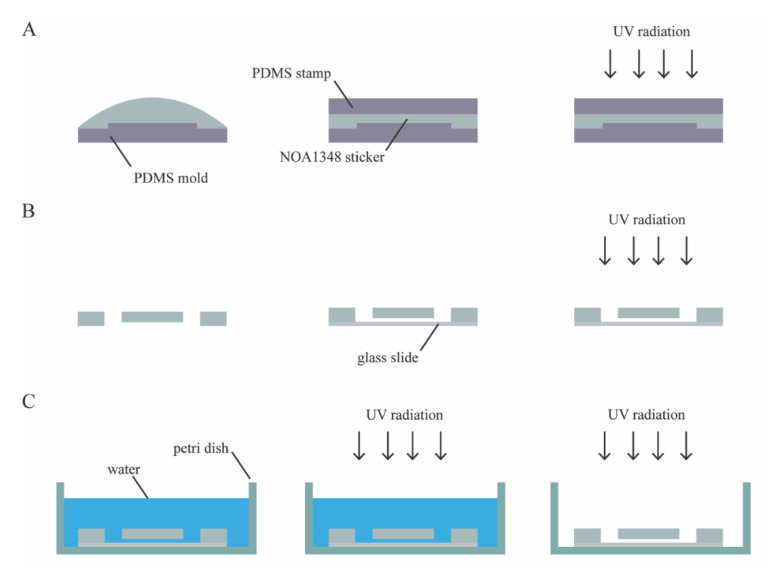
Microfabrication workflow. (**A**) NOA1348 microchannel is cast using a polydimethysiloxane (PDMS) stamp and partially cured using UV radiation. (**B**) Holes are punched for the reservoirs, and the microchannel is pressed against a glass substrate and cured again. (**C**) The microchannel assembly is submerged for a final curing step; then, it is removed from water and exposed to UV radiation to sterilize microchannel and to make it more hydrophilic to improve wetting.

The microfluidic device consists of a single layer of NOA1348 (Norland Optical Adhesives, Cranbury, NJ, USA) bonded to a glass slide, as shown in Figure 1C. To make the microchannel, we pipetted 100 μL of NOA1348 directly onto the PDMS mold, as shown in Figure 2A. Then, the NOA1348 is degassed for approximately 20 min until there are no bubbles left to ensure that the traps fully develop when cured. We used a clean piece of PDMS to stamp the NOA1348 while not introducing any bubbles. After flattening the uncured material, we put the mold in the UV oven (Uvitron International, West Springfield, MA, USA), set the power to 200 W, and cure for only 10 s. This allowed the traps to develop fully, while simultaneously leaving behind a thin uncured polymer layer similar to a previously described technique [12,22]. After this short curing step, we peeled the flat piece of PDMS off of the NOA1348 prior to removing the sticker from the mold. Then, we assembled the device, as shown in Figure 2B. We placed the sticker on a self-healing mat and punched holes for reservoirs using a biopsy punch to make holes for reservoirs. We placed the sticker on a glass slide cleaned with acetone with the channel side facing down. We pressed hard on the microchannel to make sure there are no air gaps between the device and the glass substrate and then placed it back in the UV oven to cure for 100 s at 200 W. Figure 2C shows the final curing step and sterilization of the device. To finish curing, we submerged the assembled microchannel in water to prevent any inhibition of the polymerization reaction due to oxygen [22] and then cured the submerged microchannel for 100 s. To prepare the device for cell culture, we put the microchannel in a cell culture dish and wrapped it in parafilm to seal it. Then, we put it back in the UV oven for 3 h, at 400 W, to completely sterilize the microchannel. This step served the dual purpose of sterilizing the microchannel and modifying the surface chemistry of the NOA1348 to make it more hydrophilic so that water fully wets the features inside the microchannel.

### 2.6. Imaging Method

After filling the microchannel with cell culture media, we used differential phase contrast (DPC) QPI to measure the optical volume of the traps every 5 min for 2 h. DPC QPI is a technique that uses asymmetric illumination to capture the phase gradient of a transparent sample [27,28]. This was implemented on a standard compound microscope by removing the illumination source and replacing it with a programmable 8 × 8 red LED array (Adafruit, New York City, NY, USA) driven by an Arduino Metro M4 (Adafruit, New York City, NY, USA). We illuminated the sample using opposite halves a circle to obtain two images, from which the phase gradient can be determined using the equation [28,29]:

(1) $$I_{DPC}=\frac{I_{R}-I_{L}}{I_{R}+I_{L}}$$

This was repeated for both orthogonal directions from which the phase shift can be determined to measure the phase shift of light as it passes through transparent materials such as the microchannel structures or through cells.

### 2.7. Optical Volume and Mass Determination Using DPC

The measured phase shift represents the optical thickness of the transparent material. This was used to measure the optical volume by integrating over the area of the object [13]:

(2) $$V_{optical}=\int^{\;}\phi \lambda dA$$

where *ϕ* is phase shift as a fraction of a wavelength, *λ* is wavelength, and *A* is the area of the object. We validated this approach by comparing it to the theoretical optical volume of the cylindrical cell traps, which can be determined using the equation:

(3) $$V_{optical}=\;\pi r^{2}h\Delta n$$

such that *r* is the radius of the cylinder, *h* is the height of the trap, and Δn is the difference between the refractive index of the material and the refractive index of solution in the microchannel. The cell mass was measured likewise by integrating the phase shift over the area of the cell and multiplying by the known relationship between the measured phase shift and cell mass as previously described [13]:

(4) $$m=\;\frac{1}{\alpha }\int^{\;}\phi \lambda dA$$

The conversion factor, α, is known as the specific refractive increment, and it is the slope of the linear relationship between the refractive index and biomolecule concentration inside a cell, d*n*/d*c*, which is accurate within about 5% for most cells [13]. The value of α used in this study is $1.8\times 10^{-4}$ m^3^/kg, and the wavelength of the LED array we used is $624\times 10^{-9}$ m.

### 2.8. Refractive Index Measurement Using Abbe Refractometer

To validate the refractive index for our particular lot of NOA1348, we aliquoted a small amount onto the refractive prism of an Abbe refractometer. The refractometer uses an illuminating prism with a coarse edge to illuminate the sample from all angles ranging from 0 to 90 degrees. This allows the refractive prism to collect all the light over the range of illumination angles. The measurement was made by tuning the refractometer to find the critical angle at which total internal reflection occurs inside the refractive prism [30]. This measurement was used in order to determine the refractive index of the sample relative to that of the refractive prism.

### 2.9. Refractive Index Measurement Using DPC

To validate the difference in refractive index between the microchannel fabrication material and the aqueous solution inside the channel, we fabricated a microchannel using the procedure described in Figure 2. Then, we filled the device using negative pressure applied to the outlet to pull water into the microchannel, and then measured the phase shift using the imaging method described above. The measured phase shift is directly related to the refractive index of the material using the equation [13]:

(5) $$\Delta n=\;\frac{\phi \lambda }{h}$$

such that *ϕ* is the measured phase shift as a fraction of a wavelength, *λ* is wavelength, and h is the height of the microchannel. We used this measurement to validate that the measured phase shift is consistent with the refractive index of the material measured using the Abbe refractometer.

### 2.10. Image Processing

To process the images, we constructed a periodic mask outlining trap locations. This mask was computed by measuring the location of three traps in the array to account for any rotation of the microchannel and the known distance between the traps, then creating a repeating diamond pattern of areas in which phase shift (and cell mass) are computed. We used the computed mask to measure the optical volume of the empty trap locations to compute the mean optical volume of the traps in the array. We subtracted the mean optical volume of empty traps from the trap locations containing cells to obtain the optical volume of the cells. We used the optical volume of each cell to compute its mass using the specific refractive increment, which is the known relationship between a cell’s refractive index and its density [13,31].

### 2.11. Cell Culture

We cultured MCF7 cells (ATCC, USA) using Dulbecco’s Modified Eagle Medium (DMEM) (ThermoFisher, Waltham, MA, USA) supplemented with 10% fetal bovine serum (Omega Scientific, Tarzana, CA, USA) and 5% penicillin/streptomycin antibiotic supplement (ThermoFisher, Waltham, MA, USA). To prepare the microchannel for cell culture, we first sterilized it using UV radiation for 3 h with the power set to 400 W. After sterilizing, the microchannel was first filled with a 70% ethanol–water solution to eliminate bubbles from the microchannel. The ethanol–water solution in the reservoir was slowly diluted with sterile water before being flushed entirely with water to clean the ethanol out of the microchannel. Then, the water was flushed out of the microchannel using Dulbecco’s Phosphate Buffered Saline (DPBS) before injecting a 0.01% solution of poly-l-lysine into the channel. We allowed the microchannel to incubate with the poly-l-lysine for 1 h at 37 °C before seeding cells. Cells were washed with DPBS (ThermoFisher, Waltham, MA, USA), tryspsinized to remove them from the plate, counted, and diluted to a final volume of 10,000 cells/mL prior to injecting them into the microchannel device at a flow rate of 1 μL/min.

### 2.12. Biocompatibility Assay

We cured 30 μL of NOA1348 in cell culture treated 6-well plates and then washed them with 100% ethanol. Then, we washed out the ethanol with deionized water and rinsed with DPBS prior to seeding cells. For the poly-l-lysine condition, we incubated the poly-l-lysine in the appropriate wells for 1 h at 37 °C to immobilize on the substrate. Then, we seeded $3\times 10^{4}$ cells in each well and allowed them to adhere for 24 h prior to counting. Then, we counted 3 wells of each condition every 24 h to measure proliferation each over 4 days, after which cells became confluent in each well.

### 2.13. Statistics

We used box and whisker plots generated in Matlab (Mathworks, Natick, MA, USA) to show the distribution of the raw data collected in this study. The red line shown in the boxplots shows the median of the data, and the blue box shows the data that falls within the interquartile range, such that the middle 50% of the data is contained inside the blue boxes. The 75th percentile is at the top of the box, and the 25th percentile is shown as the bottom of the box. The whiskers show the maximum and minimum data points that are not considered outliers. Outliers are defined as data points that are greater than 1.5 times the interquartile range either above the 75th percentile or below the 25th percentile data points. Outliers are represented by red ‘+’ symbols on the plots.

To summarize the data, we used a measure called the coefficient of variation (CV). The CV is defined as the standard deviation divided by the mean and is used to summarize data spread relative to the mean. The CV is expressed as a percentage such that a low CV represents closely grouped together data points and a high CV represents data that is more spread out.

## 3. Results

### 3.1. Device Design and Fabrication

Our test device consists of an array of 150 circular hydrodynamic cell traps (7.5 μm radius) arranged within a microchannel chamber (Figure 1). Hydrodynamic cell traps have been used for a variety of cell analysis methods including DNA damage analysis [32] and single cell RNA-sequencing [33]. By maintaining the position of cells for long periods, traps enable repeatable measurements of cell behavior. However, the operation of cell traps requires that cells be in direct contact with the microfluidic material, leading to potential measurement artifacts. Therefore, this device design is ideally suited to the advantages of index-matched materials for microfluidics.

Our fabrication approach is depicted in Figure 2. A key step is the use of a PDMS mold to allow oxygen to partially inhibit the UV-induced polymerization reaction as in previous work on microfluidic stickers [22]. This leaves an uncured layer that can be used to create a permanent bond between the device and substrate. This technique has a major advantage over previous techniques applied to low refractive index polymers because it is the first approach to demonstrate an effective approach to bond index-matched microfabricated devices to a glass slide.

Since this technique has no reliance on mechanical force, there is also no opportunity to create pressure points that might break the glass substrate, allowing coverslips to be used as well as glass slides. Previously developed techniques use rigid materials and screws to create a clamp that provides the mechanical force to seal the microchannel against the substrate [17]. Additionally, the use of an opaque mechanical clamp with a narrow aperture limits the available angles of illumination [17]. In contrast, the present method based on a permanent bond to a glass substrate results in a thin device, enabling precise optical measurements inside the microchannel.

### 3.2. Device Validation

#### 3.2.1. Validation of Microchannel Properties

To accurately quantify the refractive index of the NOA1348 material, we first measured the refractive index of the polymer using an Abbe refractometer. We measured this optical property of the material to account for small lot-to-lot variations in the physical properties of the polymer. Using this tool, we determined that the refractive index of the uncured NOA1348 polymer to be 1.352, which is within 0.5% of the value reported by the manufacturer.

**Figure 3:**
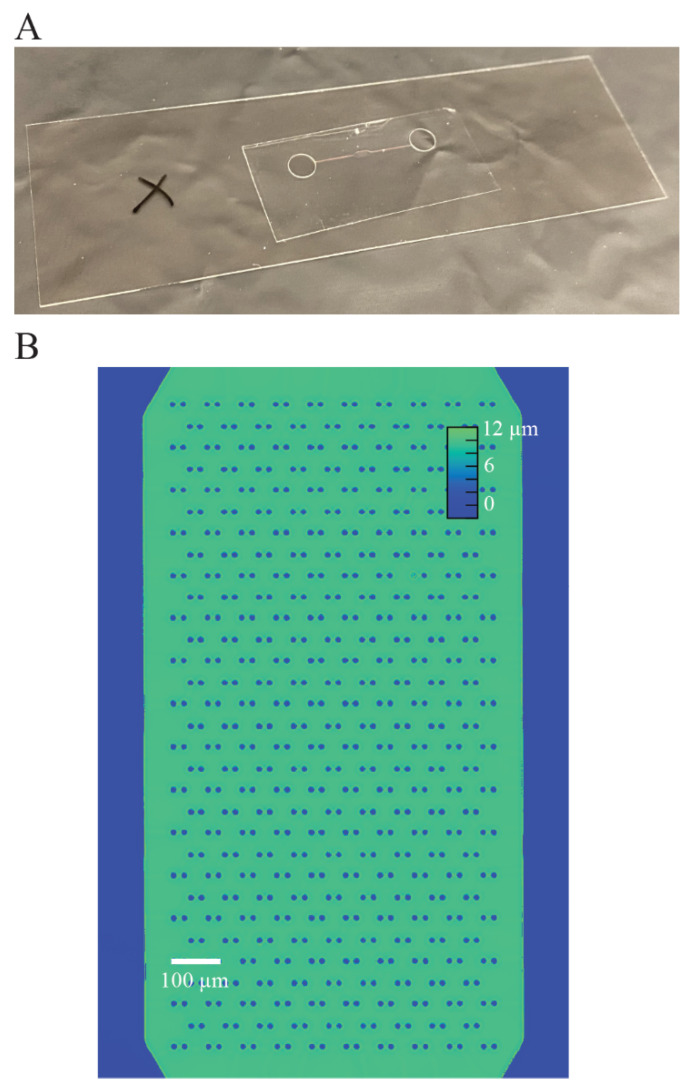
Device geometry. (**A**) Photograph of an assembled NOA1348 microfluidic sticker mounted on a #1.5 cover glass. The black ‘X’ on the slide (left) is used as an autofocus target. (**B**) Height map of circle trap array as measured using an Olympus LEXT scanning confocal microscope. These data show that the mean height of the microchannel is 9.8 μm.

Then, we measured the refractive index difference between NOA1348 and a water-filled microchannel to demonstrate the accuracy of phase measurements using DPC QPI. First, we filled an NOA1348 microchannel with deionized water and used QPI to measure the phase shift. For a uniform material, the phase shift, or optical thickness, is equal to tΔn the thickness of the material, t, times the difference in refractive index between the two materials, Δn [13]. In this case, the thickness is the channel depth, 9.8 μm as shown in the height map measured in Figure 3B using an Olympus LEXT confocal scanning microscope. The measured difference in refractive index between the water inside the microchannel and the bulk polymer material is Δn = 0.0241. Adding this to the well-established refractive index of water results in a measured refractive index of *n* = 1.354, within 0.1% of the measured value for uncured polymer using the Abbe refractometer. We validated the lateral dimensions of the device using brightfield microscopy to confirm accurate feature reproduction. The measured width of both the PDMS and NOA1348 microchannels were 50 ± 0.5 μm, which is consistent with the SU-8 master.

#### 3.2.2. NOA1348 Biocompatibility

**Figure 4:**
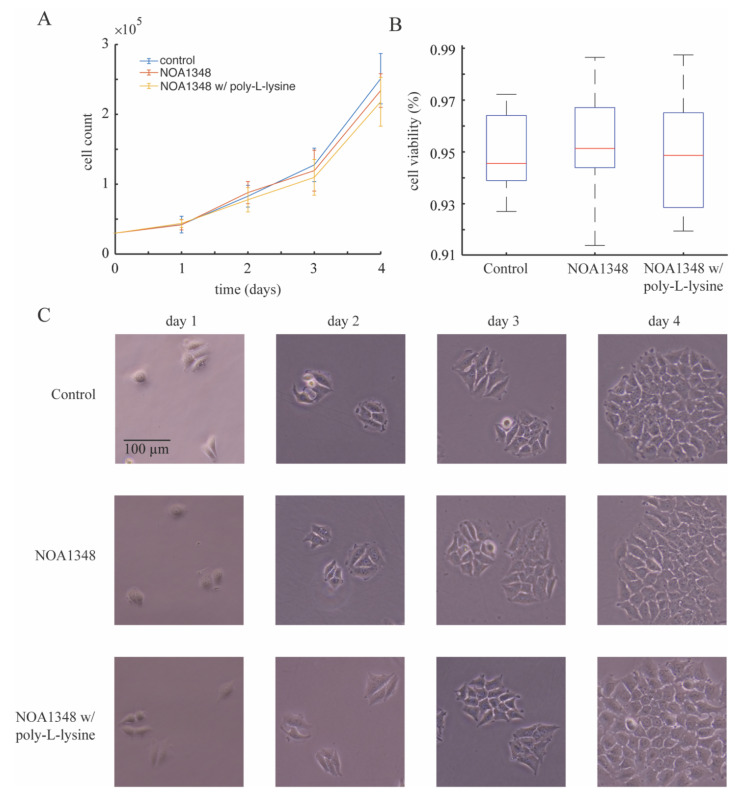
Biocompatibility of NOA1348. (**A**) Proliferation data show robust growth over the course of 4 days at a similar growth rate as the control. (**B**) Cell viability data on day 4 indicate that cells growing near NOA1348 structures show viability that is indistinguishable from the control. (**C**) Photographs of cells in all three conditions demonstrate cell colonies growing near NOA1348 grow in size similar to control conditions.

To validate the biocompatibility of this material, we cultured cells with 30 μL of cured NOA1348 with and without poly-l-lysine coating in a 6 well plate and compared this to a control that did not have any NOA1348 in the dish. We found that after four days of proliferation, as shown in Figure 4A, the cells have grown to a similar population size. Cells growing with NOA1348 had a doubling time of 32 ± 5 h (33 ± 4 h with poly-l-lysine coating)—this time is similar to the control, which grew with a doubling time of 31 ± 4 h. As shown in Figure 4B, we also measured cell viability and found that after 4 days of growth, the viability between cells in each group were indistinguishable from the control. To visualize this further, we imaged colonies of cells from each condition using phase contrast microscopy, as shown in Figure 4C. These data also indicate biocompatibility based on similar size and morphology across all three conditions.

#### 3.2.3. Validation of Trap Optical Volume

**Figure 5:**
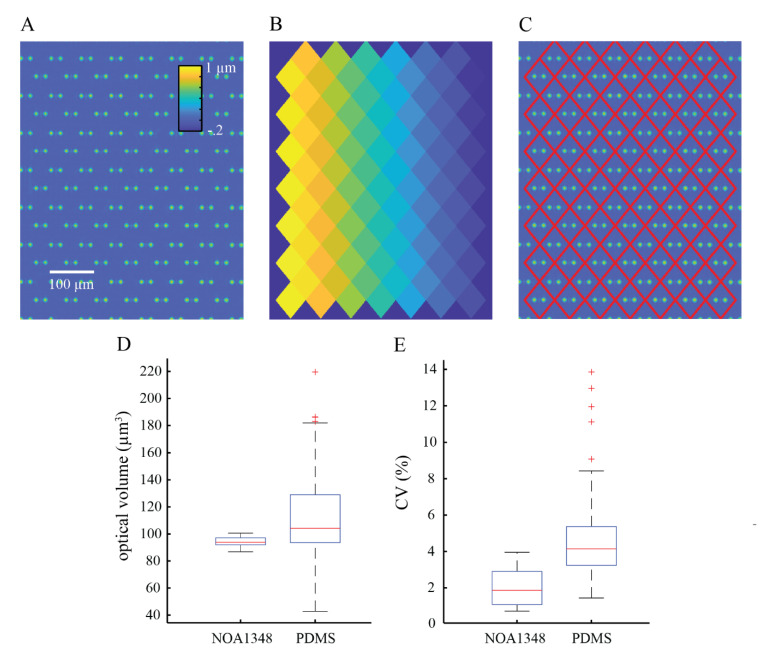
Spatiotemporal reliability of device. (**A**) Precomputed mask used for isolating cells caught in traps. (**B**) Quantitative phase imaging (QPI) measurement of empty cell traps used to validate the repeatability of the measured optical volume of each trap. (**C**) Masked trap image using the mask in panel A and the image in panel B to isolate each trap from one another for independent optical volume measurements. (**D**) Boxplot showing the distribution of measured optical volumes of NOA1348 and PDMS cell traps, the median optical volume for NOA1348 traps is 98 μm^3^ only 1% greater than expected, and a spatial coefficient of variation (CV) of 4.2%. The PDMS traps have a median optical volume of 105 μm^3^, which is 64% lower than expected. The spatial CV of this data is 24.2%. (**E**) Boxplot showing the temporal precision of trap optical volume measurements of NOA1348 and PDMS. The data show that the NOA1348 has a temporal CV of 1.8% and PDMS has a temporal CV of 4.1%. Outliers are represented by ‘+’ symbols on boxplots in D and E.

Next, we validated the hydrodynamic trap optical volume using DPC QPI to measure the phase shift as light passes through the microchannel structures (Figure 5A). Typically, when obtaining QPI measurements of live cells, the image processing workflow of segmenting and tracking each cell throughout an experiment is an important source of error [34]. There are three critical image processing steps: (1) images are corrected for background phase shifts, (2) then, these images are segmented to separate cells from background, and (3) cells are tracked from frame to frame to account for motion throughout the live cell experiment [34,35,36,37,38]. Each of these steps introduces errors that compound on each other, often resulting in ambiguities in the measured mass and cell behavior. The optical properties of NOA1348 allow us to employ a novel image processing scheme that eliminates the need for a unique cell segmentation of each image in order to eliminate errors that arise during this traditional image processing workflow. We first performed a background correction by finding the mean background in an arbitrarily large area where there are no traps resulting in the image shown in Figure 5A. This eliminates the need for preliminary segmentation of cells from empty regions to find this background phase shift. To eliminate the need to segment cells from the background, we used the location of three traps in the trap array to compute a premade mask, as shown in Figure 5B,C. This mask is static and unaffected by changes in cell morphology, eliminating another important source of image segmentation noise. Then, we use this mask to isolate each trap in the field of view so we could compute the optical volume at each location. Finally, the position of each cell is fixed in place by the hydrodynamic traps, eliminating errors due to tracking.

We used QPI to measure the phase shift as light passed through the microchannel structures and the water inside the channel to measure the integrated optical thickness of the traps. Then, we integrated over the area of the measurement to quantify the optical volume of the traps. We measured the accuracy of our system to validate that our optical measurements are consistent with the predicted optical volumes of the traps based on their geometry and measured refractive index of the material. Figure 5D shows that we found that the median optical volume of the traps is 98 μm^3^, which is approximately 1% greater than the theoretical optical volume of 97 μm^3^ based on the measured optical properties of NOA1348 and the refractive index of water at 37 °C [39].

In addition to quantifying the accuracy of our system, it is equally important to measure the spatiotemporal precision of this system as well. We validated the spatial precision of our microfluidic platform by measuring the optical volume of the cell traps and showing that we obtain a similar measurement for each trap independent of their location in the field of view. This validates that our optical measurements are not biased by the setup of the system so that we can readily separate the optical volume of the traps from that of the cells in order to precisely determine the mass of cells caught in the traps. To measure how repeatable the measurement is across traps in the field of view, we computed the CV of this measurement and found the median CV to be 4.2% (Figure 5D).

Temporal repeatability is key for generating low noise measurements of cell growth by making repeated measurements of cell mass over time with QPI. To measure the temporal stability of the system, we imaged our device for 6 h, obtaining a measurement every 5 min. We used a home-built autofocus to prevent the measured optical volume from drifting over time as a consequence of thermal drift. In between optical volume measurements, the microscope would image an autofocus target and compute a focus measure based on how far the microscope has drifted since the previous measurement. The autofocus target used for these experiments was a deep scratch made using a diamond scribe when working with glass slides or an ‘X’ drawn with an ethanol-resistant pen (as shown in Figure 3A) for coverslip glass. We computed the mean optical volume for each frame. Using this setup, we measured a CV of 1.8% for the temporal repeatability of this system, as shown in Figure 5E.

We also measured the optical volume of the same trap design cast using PDMS to compare the results from the NOA1348 to a standard material used in most microfabrication workflows. PDMS has a much larger refractive index than water relative to NOA1348. Due to this large difference in optical properties, we expected that a quantitative microscopy technique such as QPI will not be able to accurately or precisely measure the optical volume of the traps as previously described for two other distinct QPI imaging methods [12]. Figure 5E shows that the measured mean optical volume of the PDMS traps is 105 μm^3^. This is 64% lower than the expected value of 290 μm^3^ and indicates that there are large errors in computing the phase shift of PDMS traps with QPI. Along with this inaccuracy, there was high variation in trap optical volume from trap to trap within the array, which we call spatial precision, resulting in a CV of 24.2%. This is significantly larger than the measured CV for NOA1348 and indicates that the large difference in refractive index between PDMS and water makes a consistent measurement of trap optical volume unreliable when using employing this technique with PDMS. Overall, this indicates that the novel image processing workflow demonstrated with the NOA1348 cell traps would not be compatible with traps made from PDMS.

### 3.3. Cell Mass and Growth in NOA1348 Microchannels

**Figure 6:**
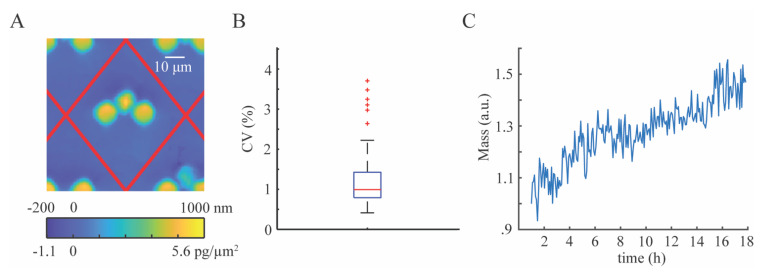
Cell growth in the NOA1348 device. (**A**) Example of image with cell caught in a trap. (**B**) Boxplot shows that the optical properties of NOA1348 combined with our image processing approach precisely measures the mass of individual cells over short periods of time. Median coefficient of variance is 1%. (**C**) Mass over time plot demonstrates MCF7 cells growing robustly when trapped in an NOA1348 microchannel. The doubling time for these cells is 32 h.

Finally, we demonstrated the use of our devices to make repeated, high-precision measurements of cell mass. Our approach using NOA1348 cell traps allows us to pin cells to a single location throughout the course of an experiment. Furthermore, the optical properties of NOA1348 enable us to employ a novel image processing approach such that we can automatically mask out the cells and traps together, allowing us to subtract the optical volume of the traps from the combined optical volume of the cell and trap so that we can isolate the optical volume of the cell as part of measuring its mass at each point in time. Therefore, we do not need to segment cells from traps or background. Since the average phase shift in the background is, on average, near zero, we are able to compute the optical volume of the entire region surrounding the trap and obtain only the optical volume of the trap and the cell combined, as shown in Figure 6A. Due to the high spatial repeatability of the traps in the array, the mean optical volume of the traps can be subtracted from the computed cell-trap optical volume in order to obtain the optical volume of the cell. This allows us to readily compute the mass of trapped cells, with high temporal repeatability. Figure 6B shows that on average, we found the coefficient of variation for the measurement of individual cells to be 1%. To demonstrate the application of this material and device for measuring cell growth, we measured the mass of MCF7 cells over time, as shown in Figure 6C. We measured the mass of each cell using DPC QPI every 5 min for 18 h. The mass of each cell is normalized by its initial mass and averaged at each time point with each other cell trapped in the array. The exponential growth rate of these cells is 0.021 h^−1^, representing a doubling time of 32 h, which is consistent with proliferation assay results (Figure 3), and validating the use of this device to measure cell growth with QPI.

## 4. Discussion

We have shown that NOA1348 (*n* = 1.352) can be used as a viable alternative to traditional microfluidic fabrication materials such as glass, silicon, or PDMS. NOA1348 is a UV-curable polymer that is compatible with most soft lithography techniques, making it readily integrated into the microfabrication workflows currently employed in most laboratories. The most important feature of this material is its ability to enhance the precision of optical readouts in microfluidics applications, employing the use of aqueous solutions (*n* = 1.33) due to its low refractive index relative to traditional materials such as PDMS (*n* = 1.43). This becomes especially important when objects inside the microchannel are in close proximity to microchannel structures such as cell traps. We demonstrated the utility of this material for this application using cells in aqueous cell culture media.

In this work, we demonstrated an improved method to fabricate NOA1348 devices by directly bonding to a glass substrate to attach the microchannels to a glass slide. This is an important advantage over existing techniques for working with fluorinated polymers, which often require the use of mechanical force, often creating pressure points for the glass to break [17,18]. This made these techniques incompatible with glass coverslips, which readily break under even small amounts of stress. We tested the technique demonstrated here on both glass microscope slides and #1.5 cover glass to demonstrate the wide compatibility of this approach with brittle substrates such as these because it readily adheres directly to a glass substrate.

Another important alternative approach is to use a UV-curable glue to laminate the microfluidic device to a glass substrate [15]. One important drawback to using a UV-curable adhesive to laminate the microchannel is the risk of clogging the microchannel if the adhesive is improperly applied. A microchannel that is too short or an adhesive layer that is too thick increases the likelihood of clogging to occur. Our approach mitigates this problem by using a thin layer of uncured polymer that readily adheres to a glass slide during a final cure step.

To obtain the best results using this approach, we recommend closely matching the optical path length through the microchannel and the aqueous solution. Since the optical path length difference is the product to both the refractive index difference and the height of the microchannel, we recommend the minimum microchannel height that does not obstruct the flow through the microchannel. This not only reduces artifacts near the interface between the microfabricated structures and objects inside the microchannel but also increases the precision of optical volume measurements in the microfluidic device.

We validated the performance of our index-matched cell trapping device (Figure 6) using a DPC QPI microscope, which measures the phase shift as light passes through a sample using many angles of illumination [28,40]. This gives strong evidence that our fabrication approach should be compatible with other microscopy techniques using wide angles of illumination such as Fourier ptychography, which uses illumination outside the range of the numerical aperture of the objective lens in order to increase spatial resolution and obtain 3D phase information [41].

This approach can be more broadly applied to microfluidics applications than just aqueous applications. Specialty manufacturers develop a wide range of polymers with varying optical properties, allowing them to be useful for applications requiring a wide range of common refractive indices. For example, Norland Optical Adhesives also makes a polymer that would be ideal for dimethylsulfoxide based applications (Δn = 0.019) such as for the formation of doxorubicin-loaded nanoparticles for controlled drug delivery [42]. Previous work has demonstrated how some of these materials can be permanently bonded to a glass substrate [12,22]; however, our work is the first to demonstrate a reliable fabrication method for the use of NOA1348. Furthermore, the unique optical properties of this material allow us to employ a novel image processing approach that would otherwise be difficult to implement.

An important source of error when measuring optical readouts originating from cells is properly segmenting cells and tracking the cells over time [34]. The errors here originate from the amorphous morphology of cells, making them difficult to segment. This is further complicated due to the fact that cells are constantly moving, making it difficult to track cells from frame to frame especially when the temporal resolution between frames is low. Here, we position each cell using a cell trap array, such that cells caught in traps do not move throughout the experiment, eliminating the need to track individual cells throughout the experiment. Since the locations of cells are tied to the trap and the traps have highly spatiotemporal repeatability in measured optical volume, we are able to subtract the known optical volume of the traps from the measured optical volume of the combined cell and trap such that we can isolate the optical volume of the cell and repeatably measure its mass throughout an experiment.

Here, we have demonstrated the use of this device for repeatably measuring the mass of cells, and when used for longer imaging experiments, it can readily be used to measure cell growth. Cell traps are important for single cell analysis by separating cells from each other, making segmentation easier. This approach uses a polymer, whose optical properties provides an opportunity to remove cell segmentation from the image processing workflow eliminating errors that are often introduced by it. It also facilitates tracking the cells from frame to frame, because the cell trap array isolates single cells and the temporal stability of the trap optical volume makes it a quantity that can readily be subtracted from the optical measurements of the cells. We demonstrated this image processing approach as a highly precise way to measure cell mass over time.

## Data Availability

The data presented in this study are available on request from the corresponding author.

## Supplementary Materials

The following are available online at https://www.mdpi.com/2073-4360/13/4/496/s1, Autocad design of microfluidic devices.

## References

[1] Zhao Y., Hu X.G., Hu S., Peng Y. (2020). Applications of fiber-optic biochemical sensor in microfluidic chips: A review. Biosens. Bioelectron..

[2] Dittrich P.S., Manz A. (2006). Lab-on-a-chip: Microfluidics in drug discovery. Nat. Rev. Drug Discov..

[3] Sackmann E.K., Fulton A.L., Beebe D.J. (2014). The present and future role of microfluidics in biomedical research. Nature.

[4] Ungerbock B., Charwat V., Ertl P., Mayr T. (2013). Microfluidic oxygen imaging using integrated optical sensor layers and a color camera. Lab Chip.

[5] Chang C.W., Cheng Y.J., Tu M., Chen Y.H., Peng C.C., Liao W.H., Tung Y.C. (2014). A polydimethylsiloxane-polycarbonate hybrid microfluidic device capable of generating perpendicular chemical and oxygen gradients for cell culture studies. Lab Chip.

[6] Narayanamurthy V., Nagarajan S., Firus Khan A.a.Y., Samsuri F., Sridhar T.M. (2017). Microfluidic hydrodynamic trapping for single cell analysis: Mechanisms, methods and applications. Anal. Methods.

[7] Gu X., Lei L., Sun Y.F., Si X.Y., Wang M.D., Li F., Yang G., Yang L.M., Pan G.B., Huang W. (2019). Microfluidic diffraction phase microscopy for high-throughput, artifact-free quantitative phase imaging and identification of waterborne parasites. Opt. Laser Technol..

[8] Kotnala A., Zheng Y., Fu J.P., Cheng W. (2017). Microfluidic-based high-throughput optical trapping of nanoparticles. Lab Chip.

[9] Pallotti D.K., Amoruso S., Orabona E., Maddalena P., Lettieri S. (2015). Biparametric optical sensing of oxygen by titanium dioxide. Sens. Actuators B-Chem..

[10] Caballero D., Blackburn S.M., de Pablo M., Samitier J., Albertazzi L. (2017). Tumour-vessel-on-a-chip models for drug delivery. Lab Chip.

[11] Schneider F., Draheirn J., Kamberger R., Wallrabe U. (2009). Process and material properties of polydimethylsiloxane (PDMS) for Optical MEMS. Sens. Actuators A-Phys..

[12] Kim D.N.H., Kim K.T., Kim C., Teitell M.A., Zangle T.A. (2018). Soft lithography fabrication of index-matched microfluidic devices for reducing artifacts in fluorescence and quantitative phase imaging. Microfluid. Nanofluid..

[13] Zangle T.A., Teitell M.A. (2014). Live-cell mass profiling: An emerging approach in quantitative biophysics. Nat. Methods.

[14] Popescu G., Park K., Mir M., Bashir R. (2014). New technologies for measuring single cell mass. Lab Chip.

[15] Arayanarakool R., Le Gac S., van den Berg A. (2010). Low-temperature, simple and fast integration technique of microfluidic chips by using a UV-curable adhesive. Lab Chip.

[16] Kim Y.S., Lee N.Y., Lim J.R., Lee M.J., Park S. (2005). Nanofeature-patterned polymer mold fabrication toward precisely defined nanostructure replication. Chem. Mater..

[17] Polanco E.R., Western N., Zangle T.A. (2018). Fabrication of Refractive-index-matched Devices for Biomedical Microfluidics. J. Vis. Exp..

[18] Polanco E., Griffin J., Western N., Zangle T. (2019). Low Refractive Index Microfluidic Device Fabrication for Quantitative Phase Imaging.

[19] Kim S.H., Cui Y., Lee M.J., Nam S.W., Oh D., Kang S.H., Kim Y.S., Park S. (2011). Simple fabrication of hydrophilic nanochannels using the chemical bonding between activated ultrathin PDMS layer and cover glass by oxygen plasma. Lab Chip.

[20] Kim S.H., Yang Y., Kim M., Nam S.W., Lee K.M., Lee N.Y., Kim Y.S., Park S. (2007). Simple route to hydrophilic microfluidic chip fabrication using an ultraviolet (UV)-cured polymer. Adv. Funct. Mater..

[21] Dupont E.P., Luisier R., Gijs M.A.M. (2010). NOA 63 as a UV-curable material for fabrication of microfluidic channels with native hydrophilicity. Microelectron. Eng..

[22] Bartolo D., Degre G., Nghe P., Studer V. (2008). Microfluidic stickers. Lab Chip.

[23] Levache B., Azioune A., Bourrel M., Studer V., Bartolo D. (2012). Engineering the surface properties of microfluidic stickers. Lab Chip.

[24] Gu H., Duits M.H.G., Mugele F. (2010). A hybrid microfluidic chip with electrowetting functionality using ultraviolet (UV)-curable polymer. Lab Chip.

[25] Morel M., Bartolo D., Galas J.C., Dahan M., Studer V. (2009). Microfluidic stickers for cell- and tissue-based assays in microchannels. Lab Chip.

[26] Tadayon M.A., Chaitanya S., Martyniuk K.M., McGowan J.C., Roberts S.P., Denny C.A., Lipson M. (2019). 3D microphotonic probe for high resolution deep tissue imaging. Opt. Express.

[27] Liu Z.J., Tian L., Liu S.J., Waller L. (2014). Real-time brightfield, darkfield, and phase contrast imaging in a light-emitting diode array microscope. J. Biomed. Opt..

[28] Tian L., Waller L. (2015). Quantitative differential phase contrast imaging in an LED array microscope. Opt. Express.

[29] Mehta S.B., Sheppard C.J.R. (2009). Quantitative phase-gradient imaging at high resolution with asymmetric illumination-based differential phase contrast. Opt. Lett..

[30] Dodd L.E. (1931). Calibration of Abbe refractometer with compensating prisms, to measure refractive index for any wave length. Rev. Sci. Instrum..

[31] Barer R. (1952). Interference micorscopy and mass determination. Nature.

[32] Wood D.K., Weingeist D.M., Bhatia S.N., Engelward B.P. (2010). Single cell trapping and DNA damage analysis using microwell arrays. Proc. Natl. Acad. Sci. USA.

[33] Cheng Y.H., Chen Y.C., Lin E., Brien R., Jung S., Chen Y.T., Lee W., Hao Z.J., Sahoo S., Kang H.M. (2019). Hydro-Seq enables contamination-free high-throughput single-cell RNA-sequencing for circulating tumor cells. Nat. Commun..

[34] Aknoun S., Savatier J., Bon P., Galland F., Abdeladim L., Wattellier B., Monneret S. (2015). Living cell dry mass measurement using quantitative phase imaging with quadriwave lateral shearing interferometry: An accuracy and sensitivity discussion. J. Biomed. Opt..

[35] Huang D.A., Roy I.J., Murray G.F., Reed J., Zangle T.A., Teitell M.A. (2020). Identifying fates of cancer cells exposed to mitotic inhibitors by quantitative phase imaging. Analyst.

[36] Nguyen T.L., Polanco E.R., Patananan A.N., Zangle T.A., Teitell M.A. (2020). Cell viscoelasticity is linked to fluctuations in cell biomass distributions. Sci. Rep..

[37] Mugahid D., Kalocsay M., Liu X.L., Gruver J.S., Peshkin L., Kirschner M.W. (2020). YAP regulates cell size and growth dynamics via non-cell autonomous mediators. Elife.

[38] Fanous M., Keikhosravi A., Kajdacsy-Balla A., Eliceiri K., Popescu G. (2020). Quantitative phase imaging of stromal prognostic markers in pancreatic ductal adenocarcinoma. Biomed. Opt. Express.

[39] Bashkatov A., Genina E. (2003). Water Refractive Index in Dependence on Temperature and Wavelength: A Simple Approximation.

[40] Chen M., Phillips Z.F., Waller L. (2018). Quantitative differential phase contrast (DPC) microscopy with computational aberration correction. Opt. Express.

[41] Tian L., Li X., Ramchandran K., Waller L. (2014). Multiplexed coded illumination for Fourier Ptychography with an LED array microscope. Biomed. Opt. Express.

[42] Xu J., Zhang S.S., Machado A., Lecommandoux S., Sandre O., Gu F., Colin A. (2017). Controllable Microfluidic Production of Drug-Loaded PLGA Nanoparticles Using Partially Water-Miscible Mixed Solvent Microdroplets as a Precursor. Sci. Rep..

